# Comparative effectiveness and safety of vonoprazan/amoxicillin-based dual therapy versus quadruple therapy as second-line treatment for *Helicobacter pylori* Infection: a retrospective cohort study

**DOI:** 10.1007/s10238-025-02037-8

**Published:** 2026-01-20

**Authors:** Ying-Ying Han, Ji-Yan Li, Ya-Ni Zhou, Lin Tuo, Ge Wang, Jing-Mei Liu, Zhen-Zhen Zhou, Mei Liu, Pei-Yuan Li

**Affiliations:** 1 Division of Gastroenterology, Tongji Hospital, Tongji Medical College, Huazhong University of Science and Technology, 1095 Jiefang Avenue, Wuhan, 430030 China; 2 Health Management Center, Tongji Hospital, Tongji Medical College, Huazhong University of Science and Technology, 1095 Jiefang Avenue, Wuhan, 430030 China

**Keywords:** *Helicobacter pylori*, Second-line treatment, Dual therapy, Bismuth quadruple therapy, Vonoprazan, Amoxicillin

## Abstract

The introduction of vonoprazan has markedly enhanced the effectiveness of the first-line *Helicobacter pylori* (*H. pylori*) eradication regimens. This study aimed to compare the effectiveness of vonoprazan-amoxicillin based dual therapy with that of quadruple therapy as second-line treatments, while also investigating potential clinical predictors of therapeutic success. From January 2023 to June 2025, we retrospectively analyzed clinical data from *H. pylori*-infected patients who received second-line treatment with either: vonoprazan-amoxicillin dual therapy (VA) or VA based quadruple therapy (VAMB; vonoprazan, amoxicillin, minocycline, and colloidal bismuth pectin). The eradication status was evaluated by ^13/14^C­urease breath test four weeks after treatment completion. Adverse events and medication compliance were systematically documented during follow-up. The study included 241 patients, with 107 receiving VA dual therapy and 134 undergoing VAMB quadruple therapy. Eradication rates were comparable between groups: 90.7% (VA) versus 92.5% (VAMB) by modified intention-to-treat (mITT) analysis, and 91.4% versus 93.7% by per-protocol (PP) analysis (all *p* > 0.05). Notably, the VA regimen demonstrated significantly fewer adverse events (8.4% *vs* 17.9%, *p* = 0.033). Both treatment arms maintained excellent medication adherence. Compared to the vonoprazan-amoxicillin-minocycline-bismuth (VAMB) quadruple regimen, vonoprazan-amoxicillin (VA) dual therapy achieved comparable eradication efficacy with a more favorable safety profile in second-line *H. pylori* treatment, representing a simplified yet effective rescue therapy option.

## Introduction

*Helicobacter pylori* (*H. pylori*) infection remains a serious global health challenge, leading to various gastric and extra-gastric complications [1]. Despite declining prevalence in certain regions, recent studies indicate that over 40% of adult worldwide remain infected, imposing a substantial burden on healthcare systems [2, 3]. Successful eradication of *H. pylori* infection can help to restore normal gastric mucosa, relieve symptoms, and reduce gastric cancer risk [4]. However, rising antibiotic resistance—particularly to clarithromycin, levofloxacin, and metronidazole—has significantly compromised eradication rates [5]. Reports indicate that 10–20% of *H. pylori* infection patients failed in initial treatment and required rescue therapy [6, 7]. Repeated eradication failures further promote the development of multidrug-resistant strains, posting major challenge for subsequent treatment strategies.

Although individual susceptibility testing (molecular or after culture) is recommended for rescue therapy, such protocols are not routinely implemented in clinical practice due to their cumbersome procedures and high costs [4, 8]. The Maastricht VI/Florence consensus recommended fluoroquinolone­containing regimens as empiric second-line therapy when patients failed to first triple or non­bismuth quadruple therapies [4]. Given that the resistance rate of levofloxacin has reached 30% to 50% in China, fluoroquinolone-based regimens are not suitable as salvage treatment [5]. The 2022 Chinese guideline continued to recommend bismuth quadruple therapy (BQT) for empiric second-line treatment of *H. pylori* infection, utilizing antibiotic combinations with low resistance rates, such as amoxicillin, tetracycline, or furazolidone [8]. In contrast, minocycline—a tetracycline derivative with broad-spectrum antibacterial activity—has been used in *H. pylori* eradication for over two decades [9]. It exhibits a low resistance rate and is widely accessible in clinical settings [10]. Both minocycline and amoxicillin demonstrate high efficacy against *H. pylori* infection, and their combination offers several advantages, including simplified dosing, favorable safety, and good tolerability. As a result, this regimen has become a common empirical choice for rescue therapy [11].

Recent studies [12] have demonstrated that high-dose dual therapy (HDDT), consisting of esomeprazole or rabeprazole (double dose bid or standard dose qid) combined with amoxicillin (≥ 3 g/day, administered as 1 g tid or 0.75 g qid), has achieved satisfactory efficacy with fewer adverse reactions, making it a recommended option for both first- and second-line eradication treatments [8]. The introduction of vonoprazan, a reversible potassium-competitive acid blocker (P-CAB), has further enhanced *H. pylori* eradication rates due to its more potent and sustained acid suppression compared to proton pump inhibitors (PPIs) [13]. Our previous research established that a 10-day vonoprazan-amoxicillin (VA) dual therapy could achieve an eradication rate of over 90% in the first-line treatment [14]. Nevertheless, data supporting VA dual therapy as second-line regimen remain limited. Existing reports show considerable regional variation in outcomes, and few studies have systematically compared the efficacy and safety profiles between VA-based dual therapy and quadruple regimens in remedial settings.

To address these knowledge gaps, this study aimed to compare the efficacy and safety of VA dual therapy versus VAMB (vonoprazan-amoxicillin-minocycline-bismuth) quadruple therapy as second‑line treatments for *H. pylori* infection.

## Methods

### Study design and patients

This was a retrospective cohort study conducted at Division of Gastroenterology in Tongji Hospital, Tongji Medical College, Huazhong University of Science and Technology, Wuhan, China. Data were retrieved from consecutive patients between January 2023 and June 2025. We enrolled *H. pylori*-infected patients who failed only one course of *H. pylori* eradication, and received VA or VAMB regimen as second-line treatment. In this study, the selection of VA or VAMB regimen as second-line treatment was made by the attending physician, taking into account the patient’s previous treatment history, compliance, potential adverse reaction risks, and through shared decision-making with the patient.

Exclusion criteria encompassed: (i) naive to *H. pylori* treatment; (ii) history of gastrectomy; (iii) intake of antibiotics, bismuth, Chinese traditional medicines with antibacterial effect within the preceding 4 weeks, or PPIs, P-CAB, H_2_ receptor antagonists within the previous 2 weeks. Medical information, such as demographic information, clinical characteristics, treatment outcome, adverse effects, and compliance were extracted from the electronic medical records and telephone/WeChat (a mobile messaging application) follow-up.

### Ethics statement

This study was conducted in accordance with the Declaration of Helsinki. The study protocol was approved by the Institutional Ethics Board of Tongji Hospital, Wuhan, China (Approval Number: TJ-IRB202505047, dated May 30, 2025). All participants signed written informed consent forms before enrolment.

### Diagnosis of *H. pylori* infection and treatment regimen

*H. pylori* infection was diagnosed by positive ^13/14^C­urease breath test (^13/14^C-UBT), immuno-histochemical staining of biopsy samples, or stool antigen test (SAT). As to the outcome of treatment, the *H. pylori* status was assessed by ^13/14^C-UBT at least 4 weeks after completion of therapy.

The VA dual regimen consisted of VPZ (Takeda Pharmaceutical Co., Ltd., China) 20 mg twice daily and amoxicillin (Hainan General Sanyang Pharmaceutical Co., Ltd., China) 1000 mg three times daily (both after meals) for 14 days. The VAMB quadruple regimen consisted of VPZ (Takeda as above) 20 mg twice daily, amoxicillin (General Sanyang as above)1000 mg twice daily, minocycline (Hanhui Pharmaceutical Co., Ltd., China) 100 mg twice daily, and colloidal bismuth pectin (Heilongjiang Dilong Pharmaceutical Co., Ltd., China) 200 mg twice daily for 14 days. Patients were suggested to take vonoprazan and colloidal bismuth pectin half an hour before meals, while amoxicillin and minocycline taken immediately after meals.

### Study outcomes

The primary outcome was the eradication rate. Patients who were lost to follow-up or lacked re-examination results were excluded before the modified intention-to-treat (mITT) analysis, and those with poor compliance were further excluded before the per-protocol (PP) analysis. The secondary outcomes included the incidence of adverse events, compliance, and related factors might affect the therapeutic effect. Adverse events were reported by participants as instructed according to the influence of adverse events on their daily activities. It was graded as “mild” (existing but transient, tolerable, and not affecting daily life), “moderate” (psychological or physical discomfort, partly affecting daily life), and “severe” (severe interruption of their daily activities). The frequency of adverse events was assessed by counting the number of patients with each event. Multiple adverse reactions may occur in one patient and should be counted separately. Compliance was considered as “poor”, if the patient had taken < 80% of the prescribed medications.

### Statistical analysis

Continuous variables were expressed as median (interquartile range), and were compared through the Mann–Whitney test. Categorical variables were presented as numbers and percentages (%), and were compared through the Chi-square test or Fisher’s exact test, as appropriate. Univariate analysis was performed to explore significant predictive variables. All statistical calculations were performed using IBM SPSS Statistics version 26.0 (IBM Corp., Armonk, NY, USA). *P* value with both-sided < 0.05 was regarded statistically significant.

## Results

### Baseline characteristics

**Fig. 1:**
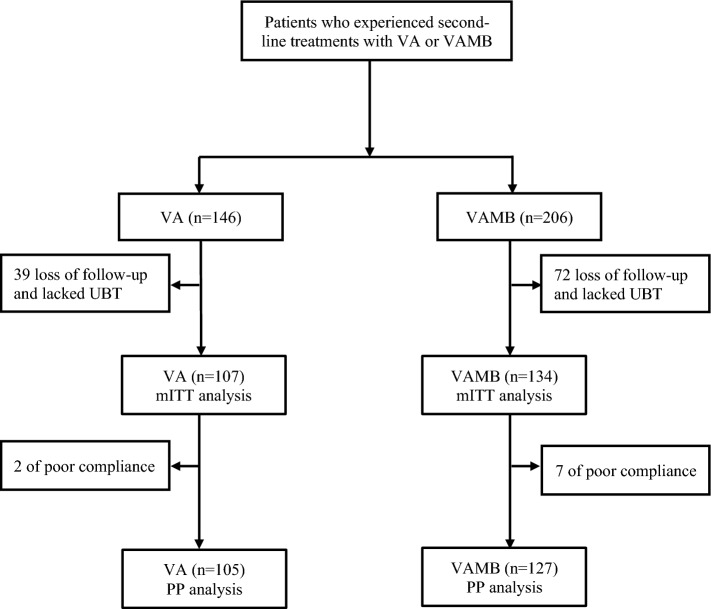
Flow diagram of this study. VA, dual therapy with vonoprazan and amoxicillin dual therapy; VAMB, bismuth quadruple therapy with vonoprazan, amoxicillin, and minocycline; mITT, modified intention-to-treat; PP, per-protocol; UBT, urea breath test

As shown in Fig. 1, a total of 352 patients with first treatment failure of *H. pylori* infection received second-line treatment with either the VA or VAMB regimens. After excluding those lost to follow-up or lacking re-examination results, 107 subjects in the VA group and 134 in the VAMB group were included in the mITT analysis. Additionally, 2 patients in the VA group and 7 in the VAMB group were excluded from the PP analysis for poor compliance.

The demographic and clinical characteristics of the mITT population are summarized in Table 1. Baseline characteristics were comparable between the two groups, with the exception of previous treatment regimens. Specifically, the proportion of patients who had received dual therapy as first-line treatment was significantly higher in the VAMB group than in the VA group (10.4% *vs.* 2.8%, *p* = 0.021).

**Table 1:** Baseline demographics and clinical data of patients

Baseline factors	VA (n = 107)	VAMB (n = 134)	*P* value
Sex (male: female)	47:60	56:78	0.739
Age (years)	46.0 (34.0–56.0)	48.5 (36.0–57.0)	0.278
BMI (kg/m^2^)	22.7 (20.5–24.7)	22.9 (21.4–24.2)	0.674
BSA (m^2^)	1.63 (1.50–1.82)	1.64 (1.54–1.78)	0.702
Family population (< 3: ≥ 3)	17:90	16:118	0.376
Style of dining (individual dining: gather dining)	48:59	51:83	0.286
Smoking	17(15.9%)	22(16.4%)	0.912
Drinking	23(21.5%)	23(17.2%)	0.395
Time to rescue therapy			0.252
< 6 months	10(9.3%)	19(14.2%)	
≥ 6 months	97(90.7%)	115(85.8%)	
Previous therapy			
BQT	73(68.2%)	87(64.9%)	0.590
Triple therapy	18(16.8%)	16(11.9%)	0.279
Dual therapy	3(2.8%)	14(10.4%)	0.021
Unknow	13(12.1%)	17(12.7%)	0.900
Amoxicillin used previously	91(85.0%)	104(77.6%)	0.145
Digestive Symptoms	57(53.3%)	64(47.8%)	0.395
Abdominal distension	24(22.4%)	26(19.4%)	0.565
Stomachache	15(14.0%)	16(11.9%)	0.632
Belching	19(17.8%)	17(12.7%)	0.273
Abdominal discomfort	5(4.7%)	7(5.2%)	0.845
Acid reflux/Heartburn	21(19.6%)	18(13.4%)	0.195
Nausea/Vomiting	3(2.8%)	3(2.2%)	1.000
Others	7(6.5%)	4(3.0%)	0.315
Endoscopy diagnosis	76(71.0%)	95(70.9%)	0.982
Non-atrophic gastritis	36(33.6%)	59(44.0%)	0.101
Atrophic gastritis	30(28.0%)	30(22.4%)	0.314
Peptic ulcer	12(11.2%)	17(12.7%)	0.727
Reflux esophagitis	4(3.7%)	9(6.7%)	0.309
Gastric polyp	5(4.7%)	3(2.2%)	0.493
Gastric cancer	1(0.9%)	1(0.7%)	1.000
Family history of gastric carcinoma	5(4.7%)	2(1.5%)	0.282

BMI, body mass index; BSA, body surface area; BQT, bismuth quadruple therapy

### *H. pylori* eradication rates

Eradication rates are presented in Table 2. In the mITT analysis, the eradication rates were 90.7% (97/107; 95% CI 85.0–96.3%) in the VA group and 92.5% (124/134; 95% CI 88.0–97.0%) in the VAMB group. Similarly, in the PP analysis, eradication rates were 91.4% (96/105; 95% CI 86.0–96.9%) and 93.7% (119/127; 95% CI 89.4–98.0%), respectively. No statistically significant differences were observed between the two groups in either analysis (mITT: *p* = 0.599; PP: *p* = 0.509).

**Table 2:** Eradication rates of the two treatment groups

Analysis	VA	VAMB	*P* value
mITT	90.7% (97/107)	92.5% (124/134)	0.599
95% CI	85.0%-96.3%	88.0%-97.0%	
PP	91.4% (96/105)	93.7% (119/127)	0.509
95% CI	86.0%-96.9%	89.4%-98.0%	

CI, confidence interval; mITT, modified intention-to-treat; PP, per-protocol

### Adverse events and compliance

Adverse events (AEs) observed during eradication therapy in both groups are detailed in Table 3. The overall incidence of AEs was significantly higher in the VAMB group (17.9%, 24/134) than in the VA group (8.4%, 9/107; *p* = 0.033). In the VA group, the most frequently reported AEs included nausea/vomiting, diarrhea, or abdominal distension. One patient experienced transient but notable abdominal pain, which resolved after treatment discontinuation. The VAMB group primarily reported dizziness, nausea/vomiting, or abdominal distension, with no treatment discontinuations due to AEs. Due to the retrospective design of this study, mild or transient AEs—such as amoxicillin-associated rashes—may not have been systematically documented. Importantly, all AEs were mild to moderate in severity and resolved spontaneously after treatment completion. Treatment compliance was excellent and comparable between groups: 98.1% (105/107) in the VA group versus 94.8% (127/134) in the VAMB group (*p* = 0.306).

**Table 3:** Adverse events and compliance of the two treatment groups

Analysis	VA (n = 107)	VAMB (n = 134)	*P* value
**Adverse effects**			
No. of patients	9 (8.4%)	24 (17.9%)	0.033
Total No. of events	12 (11.2%)	30 (22.4%)	0.023
Nausea/Vomiting	4 (3.7%)	6 (4.5%)	1.000
Dizziness	1 (0.9%)	8 (6.0%)	0.088
Diarrhea	4 (3.7%)	3 (2.2%)	0.762
Abdominal distension	2 (1.9%)	4 (3.0%)	0.892
Poor appetite	0	3 (2.2%)	0.331
Dry mouth	0	3 (2.2%)	0.331
Abdominal pain	1 (0.9%)	2 (1.5%)	1.000
Black tongue	0	1 (0.7%)	1.000
Discontinued drugs because of adverse events	1 (0.9%)	0	0.444
**Compliance**	98.1% (105/107)	94.8% (127/134)	0.306

### Factors influencing eradication efficacy

The factors influencing the eradication efficacy were presented in the mITT population. As presented in Table 4, demographic and lifestyle factors including sex, age, body mass index (BMI), body surface area (BSA), smoking status, alcohol consumption, style of dining, family population, and presence of digestive symptoms showed no significant association. Additionally, treatment-related factors such as occurrence of side effects and medication compliance were similarly not correlated with treatment outcomes (all *p* > 0.05).

**Table 4:** Univariate analysis of factors influencing eradication rates in the two treatment groups

Variables	VA (n = 107)		VAMB (n = 134)	
	Eradication rate	*P* value	Eradication rate	*P* value
Sex				
Male	91.5% (43/47)	1.000	87.5% (49/56)	0.122
Female	90.0% (54/60)		96.2% (75/78)	
Age, years				
< 35	89.7% (26/29)	0.703	95.7% (22/23)	0.087
35–50	87.9% (29/33)		98.0% (48/49)	
> 50	93.3% (42/45)		87.1% (54/62)	
BMI, kg/m^2^				
< 22	92.7% (38/41)	0.841	97.6% (41/42)	0.312
22–25	90.0% (36/40)		89.6% (60/67)	
> 25	88.5% (23/26)		92.0% (23/25)	
BSA, m^2^				
< 1.65	89.5% (51/57)	0.908	95.9% (70/73)	0.199
≥ 1.65	92.0% (46/50)		88.5% (54/61)	
Smoking				
Yes	100% (17/17)	0.323	81.8% (18/22)	0.099
No	88.9% (80/90)		94.6% (106/112)	
Drinking				
Yes	82.6% (19/23)	0.275	87.0% (20/23)	0.495
No	92.9% (78/84)		93.7% (104/111)	
Style of dining				
Individual dining	95.8% (46/48)	0.185	96.1% (49/51)	0.377
Gather dining	86.4% (51/59)		90.4% (75/83)	
Family population				
< 3	94.1% (16/17)	0.936	81.3% (13/16)	0.186
≥ 3	90.0% (81/90)		94.1% (111/118)	
Time to rescue therapy, month		0.235		1.000
< 6	80.0% (8/10)		94.7% (18/19)	
≥ 6	91.8% (89/97)		92.2% (106/115)	
Amoxicillin used previously		1.000		0.560
Yes	90.1% (82/91)		91.3% (95/104)	
No	93.8% (15/16)		96.7% (29/30)	
Digestive symptoms				
Yes	91.2% (52/57)	1.000	95.3% (61/64)	0.401
No	90.0% (45/50)		90.0% (63/70)	
Side effects				
Yes	88.9% (8/9)	1.000	91.7% (22/24)	1.000
No	90.8% (89/98)		92.7% (102/110)	
Compliance				
Good	91.4% (96/105)	0.179	93.7% (119/127)	0.149
Poor	50% (1/2)		71.4% (5/7)	

We further examined the potential impact of two specific clinical factors: the interval before rescue therapy and prior amoxicillin use in first-line treatment. The analysis revealed that neither the duration between treatments nor previous amoxicillin exposure significantly affected the eradication rate (all *p* > 0.05).

## Discussion

We conducted a retrospective cohort study to evaluate the efficacy and safety of VA dual therapy and VAMB quadruple therapy as second-line treatments for *H. pylori* infection after first-line treatment failure. Although this study was conducted in a tertiary academic hospital, which may not fully represent the broader primary care settings, it represents routine practice within a specialized referral center. The treatment decisions, patient management, and outcome assessments reflect routine clinical care without experimental intervention, preserving the essence of an observational effectiveness study [15]. Both the mITT and PP analysis yielded eradication rates exceeding 90% for each regimen as second-line therapies. No statistically significant difference in efficacy was observed between the two groups. Moreover, the VA dual therapy showed a more favorable safety profile, with a significantly lower incidence of adverse events (8.4% *vs.* 17.9%, *p* < 0.05). Eradication success was unaffected by the interval time to rescue therapy, prior amoxicillin usage, or patient compliance. Given its comparable efficacy and superior tolerability, the VA dual therapy may be considered a preferred option for second-line *H. pylori* eradication.

The antibiotic resistance remains the primary challenge in the eradication of *H. pylori* infection. A recent meta-analysis [5] has revealed alarming resistance patterns in mainland China: the primary and secondary resistance rates were 30.72% and 75.53% for clarithromycin, 32.98% and 58.17% for levofloxacin, and 70.14% and 88.56% for metronidazole, respectively; while resistance rates remained relatively low for amoxicillin (2.41% and 3.43%), tetracycline (2.53% and 0.42%), and furazolidone (1.54% and 0.45%). Despite these favorable low resistance profiles for furazolidone, tetracycline, and amoxicillin, the clinical utility of furazolidone and tetracycline is constrained by safety concerns or limited availability compared to amoxicillin, rendering the selection of appropriate antibiotic combinations for rescue therapy particularly challenging [16]. Several studies highlighted promising results with amoxicillin and minocycline-based bismuth quadruple therapy (BQT). You et al. [17] reported 92% (66/72) and 100% (42/42) eradication rates in *H. pylori* first-line and rescue therapy, respectively. A Southwest China real-world study [18] achieved 91.3% (21/23) success in *H. pylori* treatment-experienced patients. While these studies yielded promising outcomes, their interpretation was limited by relatively small sample sizes and heterogeneous patient populations with varying treatment histories. Thereby, it precluded definitive conclusions regarding VAMB’s efficacy specifically in the second-line treatment. Our study included 134 patients with precisely one prior treatment failure, demonstrating that the VAMB regimen achieved eradication rates of 92.5% (mITT) and 93.7% (PP) as a second-line therapy. Such high efficacy may be attributed to the rapid and potent acid-suppressive effect of vonoprazan, and the high sensitivity of *H. pylori* to the amoxicillin-minocycline combination, additionally with the function of bismuth.

In 2020, Suzuki et al. [19] first established the clinical efficacy of VA dual therapy for treatment-naive patients with *H. pylori* infection. Recognized for its superior efficacy, simplified dosing regimen, and favorable tolerability profile, this approach has been endorsed as a first-line therapy in the current American College of Gastroenterology (ACG) Clinical Guideline [20]. Emerging evidence supports the application of VA dual therapy in rescue treatment settings. A retrospective study [21] demonstrated an overall eradication rate of 92.5% (172/186), with subgroup analysis revealing 92.9% (78/84), 96.3% (52/54), and 87.5% (42/48) success rates for patients with 1, 2, or ≥ 3 prior treatment failures, respectively. Liu et al. [22] conducted a randomized controlled trial comparing VA dual therapy with culture-guided therapy in treatment-experienced patients, finding comparable efficacy (87.5% *vs.* 83.3%) but superior tolerability with the VA regimen. However, another study [23] reported somewhat lower success rates (ITT: 73.8%; PP: 82.1%) using the same VA regimen, though their study lacked stratification by prior treatment attempts. These outcome variations likely reflected differences in study samples, regional resistance patterns, and inclusion criteria. Our results showed that the efficacy of the VA group exceeds 90% as a second-line treatment, probably attributable to several key factors: (1) rigorous patient selection limited to first-treatment failure; (2) persistently low secondary resistance to amoxicillin (3.43% in China) [5]; (3) potent acid-suppressive effect of vonoprazan; (4) A 14-day long course of treatment can ensure the efficacy of VA dual regimen.

It is noteworthy that the dosing schedule of amoxicillin differed between the two regimens: 1000 mg three times daily in the VA group versus 1000 mg twice daily in the VAMB group. This reflects the distinct rationales behind each regimen—maximizing the time above MIC for a single antibiotic in the dual therapy [12] versus employing a standard dose within a broader-spectrum combination in the quadruple therapy [8]. Our results showed comparable eradication rates between the two groups (mITT analysis: 90.7% *vs*. 92.5%, *p* > 0.05). This suggests that in the VA regimen, the strategy of intensifying amoxicillin dosing may effectively compensate for the synergistic antimicrobial effect offered by the addition of minocycline and bismuth in the quadruple regimen.

The VA regimen demonstrated significantly better tolerability, with an AEs incidence of 8.4% compared to 17.9% of VAMB regimen (*p* < 0.05). Nausea/Vomiting and diarrhea were common adverse reactions in VA group. Dizziness and Nausea/Vomiting were more common in VAMB group, consistent with previous findings from minocycline-containing regimens [10, 24]. Owing to the higher lipid solubility and being easier to across the blood–brain barrier than other tetracyclines, minocycline may cause more neurological-related side effects [25]. The standard dosage of minocycline was 200 mg/d for the eradication of *H. pylori* infection [10]. Huang et al. [26] pioneered an optimized quadruple regimen using reduced-dose minocycline (50 mg twice daily) combined with metronidazole (400 mg thrice daily). This modified bismuth quadruple therapy maintained high efficacy while demonstrating improved safety and compliance profiles compared to conventional dosing in refractory *H. pylori* patients. However, a meta-analysis [10] reported no significant difference in adverse events between quadruple therapies with or without minocycline (RR = 0.94, *p* = 0.63). Prospective and multicenter clinical trials are needed to fully establish the efficacy and safety of low-dose minocycline-containing regimens, especially in remedial treatment.

However, univariate analysis did not identify any risk factors associated with eradication failure. The optimal interval between rescue treatments has been a subject of ongoing debate. It’s reported that *H. pylori* might enter a non-replicative, non-culturable state with low metabolic activity after repeated eradication failures [27]. Some studies proposed that delaying retreatment for over 6 months might promote bacterial reversion to an active replicative state, potentially enhancing eradication efficacy [28]. However, our findings demonstrated no significant difference in treatment efficacy whether the interval was shorter or longer than 6 months. Recently, Lin et al. [29] investigated the association between eradication rate and treatment interval duration in a prospective observational study. Patients were stratified into four groups based on retreatment intervals (1–3 months, 3–6 months, 6–12 months, and > 12 months), yet no statistically significant differences in eradication rates were observed across groups. These findings collectively suggested that extending the interval between rescue treatments might offer no discernible clinical advantage.

The selection of sensitive antibiotics remains the most critical determinant of successful rescue treatment. Our analysis revealed that patients with prior amoxicillin exposure in both groups tended to have lower eradication rates. However, this observed difference lacked statistical significance, potentially attributable to subjects with only once treatment failure, or relatively less samples. Another notable observation is that the proportion of patients who had received dual therapy as first-line treatment was significantly higher in the VAMB group than VA group (10.4% *vs.* 2.8%, *p* = 0.021). This likely reflects a common "step-up" strategy in clinical practice, wherein patients who do not respond to first-line dual therapy are generally not retreated with the same regimen, but are instead switched to a more potent quadruple therapy as a second-line option [16]. Refractory *H. pylori* infection is clinically defined as persistent infection following ≥ 2 failed standard eradication attempts in China [8]. Of particular concern in this patient population is the emergence of amoxicillin resistance, with resistance rate exceeding 10% [24]. Xie et al. [30] documented particularly high secondary resistance to amoxicillin (40%, 4/10) in certain area, which significantly compromised the effectiveness of amoxicillin-based bismuth quadruple therapy. Current evidence supports two principal approaches for refractory *H. pylori* infection: (1) drug sensitivity-guided regimens, or (2) empirical bismuth quadruple regimens containing tetracycline, minocycline, or furazolidone.

Treatment compliance remains critical for successful eradication, with both groups in our study maintaining excellent adherence rates above 95%. Our previous research [14] demonstrated that poor compliance significantly diminished the effectiveness of a 10-day VA regimen. Consequently, in clinical practice, we strongly recommend adopting a 14-day treatment course of VA regimen to optimize therapeutic outcomes.

There were certain limitations existing in this work. First, as a retrospective investigation, the absence of long-term and frequent follow-up might introduce selection bias and potential data inconsistencies. For instance, mild or transient adverse events (such as mild rashes related to amoxicillin) may not have been systematically captured. Furthermore, the high rate of loss to follow-up (111/352, 31.5%) precluded a standard intention‑to‑treat (ITT) analysis and may compromise the completeness of safety data. A detailed review of medical records and final contact notes for these patients indicated that disengagement was primarily due to logistical and behavioral factors, including symptom resolution leading to perceived cure (> 50%), geographical or practical barriers to returning for re‑examination, and non-adherence unrelated to adverse events. Importantly, there were no documented contacts, emergency visits, or patient-reported discontinuations due to suspected adverse effects during treatment or at the final follow-up. In accordance with common practice in similar studies, the mITT population was used for the primary efficacy assessment [30, 31]. The primary purpose of ITT analysis is to preserve the comparability achieved through randomization. In this present study, however, treatment allocation was guided by clinical judgment and individual patient factors rather than randomization. Thus, the mITT approach may better approximate the true clinical effectiveness of the regimen in routine practice. Nevertheless, these constraints underscore the importance of implementing systematic, outcome‑driven follow‑up protocols in prospective studies to fully capture treatment outcomes and safety profiles [32]. Second, because the study was conducted in a tertiary referral center, the findings may be most applicable to institutions managing similarly complex patient populations; generalizability to primary care settings requires further validation. Nevertheless, treatment decisions, follow‑up, and outcome evaluation in this study all followed routine clinical pathways without research‑driven intervention, thereby preserving the study’s observational validity. Third, antibiotic susceptibility testing was not performed for those rescue patients. While current guidelines recommend tailored salvage therapies based on susceptibility results, such testing remains logistically and economically inaccessible in many clinical settings. Finally, the relatively small sample sizes in certain subgroups limited our ability to detect statistically significant differences between groups. Future large-scale, multicenter randomized controlled trials are warranted to further evaluate the efficacy and safety profiles of these regimens in second-line treatment.

In conclusion, the VA dual therapy has comparable eradication efficacy compared with the VAMB therapy in retreatment patients with *H. pylori* infection, and the former exhibiting a more favorable adverse event profile. Based on these results, VA dual therapy could be regarded as the preferred second-line treatment option for patients experiencing their first *H. pylori* treatment failure.

## Data Availability

The data that support the findings of this study are available on request from the corresponding author.

## References

[1] Crowe SE. Helicobacter pylori infection. N Engl J Med. 2019;380(12):1158–65. 10.1056/NEJMcp1710945. 30893536 10.1056/NEJMcp1710945

[2] Chen YC, Malfertheiner P, Yu HT, et al. Global prevalence of Helicobacter pylori infection and incidence of gastric cancer between 1980 and 2022. Gastroenterology. 2024;166(4):605–19. 10.1053/j.gastro.2023.12.022. 38176660 10.1053/j.gastro.2023.12.022

[3] Xie L, Liu GW, Liu YN, et al. Prevalence of Helicobacter pylori infection in China from 2014-2023: a systematic review and meta-analysis. World J Gastroenterol. 2024;30(43):4636–56. 10.3748/wjg.v30.i43.4636. 39575409 10.3748/wjg.v30.i43.4636 PMC11572641

[4] Malfertheiner P, Megraud F, Rokkas T, et al. Management of Helicobacter pylori infection: the Maastricht VI/Florence consensus report. Gut. 2022. 10.1136/gutjnl-2022-327745.36113979

[5] Zeng S, Kong Q, Wu X, et al. Antibiotic resistance of Helicobacter pylori in Mainland China: a focus on geographic differences through systematic review and meta-analysis. Int J Antimicrob Agents. 2024;64(5):107325. 10.1016/j.ijantimicag.2024.107325. 39245326 10.1016/j.ijantimicag.2024.107325

[6] Li J, Shi H, Zhou F, Xie L, Lin R. The efficacy and safety of regimens for Helicobacter pylori eradication treatment in China: a systemic review and network meta-analysis. J Clin Gastroenterol. 2024;58(1):12–23. 10.1097/mcg.0000000000001902. 38084866 10.1097/MCG.0000000000001902

[7] Song Z, Suo B, Zhang L, Zhou L. Rabeprazole, minocycline, amoxicillin, and bismuth as first-line and second-line regimens for Helicobacter pylori eradication. Helicobacter. 2016;21(6):462–70. 10.1111/hel.12313. 27060292 10.1111/hel.12313

[8] Zhou L, Lu H, Song Z, et al. 2022 Chinese national clinical practice guideline on Helicobacter pylori eradication treatment. Chin Med J. 2022;135(24):2899–910. 10.1097/cm9.0000000000002546. 36579940 10.1097/CM9.0000000000002546 PMC10106216

[9] Tokunaga K, Takahashi S. Second line treatment regimen of PPI + AMPC + MNZ for patients with clarithromycin-resistant Helicobacter pylori infection. Nihon Rinsho Japanese J Clin Med. 2002;60(Suppl 2):445–8. 11979824

[10] Zhou K, Li CL, Zhang H, et al. Minocycline in the eradication of Helicobacter pylori infection: a systematic review and meta-analysis. World J Gastroenterol. 2024;30(17):2354–68. 10.3748/wjg.v30.i17.2354. 38813048 10.3748/wjg.v30.i17.2354 PMC11130572

[11] Si XB, Zhang LY, Yang S, et al. The efficacy and safety of minocycline-containing quadruple therapies against Helicobacter pylori infection: a retrospective cohort study. Infect Drug Resist. 2024;17:2513–29. 10.2147/idr.S457618. 38919832 10.2147/IDR.S457618 PMC11198024

[12] Gao CP, Zhang D, Zhang T, et al. PPI‐amoxicillin dual therapy for *Helicobacter pylori* infection: an update based on a systematic review and meta‐analysis. Helicobacter. 2020;25(4):e12692. 10.1111/hel.12692. 32314468 10.1111/hel.12692

[13] Shirley M. Vonoprazan: a review in Helicobacter pylori infection. Drugs. 2024;84(3):319–27. 10.1007/s40265-023-01991-5. 38388872 10.1007/s40265-023-01991-5 PMC11090951

[14] Han YY, Zhou L, Hu YL, et al. Comparison of vonoprazan-based with rabeprazole-based dual therapy for treatment-naive patients of Helicobacter pylori infection: a prospective, multi-center, randomized controlled study. J Gastroenterol. 2023;58(12):1167–77. 10.1007/s00535-023-02042-2. 37777987 10.1007/s00535-023-02042-2

[15] Thiese MS. Observational and interventional study design types; an overview. Biochem Med (Zagreb). 2014;24(2):199–210. 10.11613/bm.2014.022. 24969913 10.11613/BM.2014.022 PMC4083571

[16] Xu X, He C, Zhu Y. Treatment of refractory Helicobacter pylori infection: a new challenge for clinicians. Front Microbiol. 2022;13:998240. 10.3389/fmicb.2022.998240. 36329840 10.3389/fmicb.2022.998240 PMC9623003

[17] You S, Tang X, Zhou J, et al. Minocycline/Amoxicillin-based bismuth quadruple therapy for *Helicobacter pylori* eradication: a pilot study. Microorganisms. 2024. 10.3390/microorganisms12030429. 38543480 10.3390/microorganisms12030429 PMC10972041

[18] He Q, Ou Y, Zhu H, et al. Efficacy and safety of bismuth quadruple regimens containing minocycline and vonoprazan for eradication of Helicobacter pylori: real-world evidence. JGH Open. 2024;8(5):e13070. 10.1002/jgh3.13070. 38699469 10.1002/jgh3.13070 PMC11063609

[19] Suzuki S, Gotoda T, Kusano C, et al. Seven-day vonoprazan and low-dose amoxicillin dual therapy as first-line Helicobacter pylori treatment: a multicentre randomised trial in Japan. Gut. 2020;69(6):1019–26. 10.1136/gutjnl-2019-319954. 31915235 10.1136/gutjnl-2019-319954 PMC7282559

[20] Chey WD, Howden CW, Moss SF, et al. ACG clinical guideline: treatment of Helicobacter pylori infection. Am J Gastroenterol. 2024;119(9):1730–53. 10.14309/ajg.0000000000002968. 39626064 10.14309/ajg.0000000000002968

[21] Gao W, Teng G, Wang C, Xu Y, Li Y, Cheng H. Eradication rate and safety of a “simplified rescue therapy”: 14-day vonoprazan and amoxicillin dual regimen as rescue therapy on treatment of Helicobacter pylori infection previously failed in eradication: a real-world, retrospective clinical study in China. Helicobacter. 2022;27(5):e12918. 10.1111/hel.12918. 35877765 10.1111/hel.12918 PMC9542484

[22] Liu YX, Liu HN, Liu HQ, et al. Vonoprazan-Amoxicillin dual therapy versus drug sensitivity-based individualized therapy as a rescue regimen for Helicobacter pylori infection: a multicenter, randomized controlled trial. Helicobacter. 2025;30(1):e70009. 10.1111/hel.70009. 39996433 10.1111/hel.70009

[23] Zhang J, Wang X, Song S, et al. Efficacy and safety of vonoprazan and high-dose amoxicillin dual therapy for rescue treatment of *Helicobacter pylori* infection: a multicenter randomized controlled trial. United Eur Gastroenterol J. 2025. 10.1002/ueg2.70070. 10.1002/ueg2.70070 PMC12529005 40543051

[24] Huang Y, Chen J, Ding Z, et al. Minocycline vs. tetracycline in bismuth-containing quadruple therapy for *Helicobacter pylori* rescue treatment: a multicentre, randomized controlled trial. J Gastroenterol. 2023;58(7):633–41. 10.1007/s00535-023-01991-y. 37042991 10.1007/s00535-023-01991-y

[25] Smith K, Leyden JJ. Safety of doxycycline and minocycline: a systematic review. Clin Ther. 2005;27(9):1329. 10.1016/j.clinthera.2005.09.005. 16291409 10.1016/j.clinthera.2005.09.005

[26] Huang Y, Qiu S, Guo Y, et al. Optimization of minocycline-containing bismuth quadruple therapy for *Helicobacter pylori* rescue treatment: a real-world evidence study. Helicobacter. 2024;29(5):e13138. 10.1111/hel.13138. 39306798 10.1111/hel.13138

[27] Ierardi E, Losurdo G, Mileti A, et al. The puzzle of coccoid forms of *Helicobacter pylori*: beyond basic science. Antibiotics. 2020. 10.3390/antibiotics9060293. 32486473 10.3390/antibiotics9060293 PMC7345126

[28] Xie J, Liu D, Peng J, Wu S, Liu D, Xie Y. Iatrogenic factors of *Helicobacter pylori* eradication failure: lessons from the frontline. Expert Rev Anti-infect Ther. 2023;21(4):447–54. 10.1080/14787210.2023.2181788. 36794349 10.1080/14787210.2023.2181788

[29] Lin M, Hu J, Liu J, et al. The interval of rescue treatment does not affect the efficacy and safety of *Helicobacter pylori* eradication: a prospective multicenter observational study. Chin Med J. 2025;138(12):1439–46. 10.1097/cm9.0000000000003534. 40304303 10.1097/CM9.0000000000003534 PMC12180818

[30] Xie J, Peng J, Wu S, et al. Efficacy and safety of tetracycline vs. amoxicillin in furazolidone-based rescue therapy for *Helicobacter pylori*: a real-world analysis. Ann Med. 2025;57(1):2464938. 10.1080/07853890.2025.2464938. 39950212 10.1080/07853890.2025.2464938 PMC11834778

[31] Tepes B, Kastelic M, Vujasinovic M, et al. *Helicobacter pylori* treatment results in Slovenia in the period 2013-2015 as a part of European registry on *Helicobacter pylori* management. Radiol Oncol. 2018;52(1):1–6. 10.1515/raon-2017-0055. 29520199 10.1515/raon-2017-0055 PMC5839075

[32] Ma H, Zhao XH, Zhang LL, Wu LM. Application of WeChat platform in the management of patients infected with *Helicobacter pylori*. Helicobacter. 2021;26(5):e12832. 10.1111/hel.12832. 34231948 10.1111/hel.12832

